# Follow-up survey of general practitioners in Zhejiang Province post-completion of position transition training in 2017–2020

**DOI:** 10.1186/s12909-023-04151-1

**Published:** 2023-03-24

**Authors:** Hongli Qin, Shuai Li, Juanjuan Liu, Jingjing Ren, Meiyue Yu

**Affiliations:** 1 Department of General Practice, The First Affiliated Hospital, Zhejiang University School of Medicine, Hangzhou, 310003 China; 2 Zhejiang University, Hangzhou, 310003 China

**Keywords:** General practitioner, Position transition training, Follow-up survey

## Abstract

**Background:**

Position transition training for general practitioners in Zhejiang Province started in 2017 and has since been held once a year. By the beginning of 2022, four training sessions were completed. The purpose of this survey was to establish the current situation of trainees after their graduation and provide reference for the evaluation of the training effect.

**Methods:**

Of the 738 trainees who completed the training, 253 were contacted and followed up. A self-designed questionnaire was used to conduct the survey through online filling in. The content included questions to elucidate the following information: whereabouts after the training, registration as a general practitioner, undertaken general practice teaching and scientific research work, current occupational environment, improvement of post competence after receiving position transition training, willingness to complete survey, willingness to participate in future training programs, etc.

**Results:**

A number of 253 valid questionnaires were collected with a recovery rate of 100%. Notably, 93.68% of the participants successfully completed their training and obtained the Training Certificate of General Practitioners. Further, 83.4% were registered as general practitioners, 82.94% of which added on the basis of the original registered scope of practice. Currently, most of them work in primary health care institutions, primarily occupied with medical treatment, chronic disease management, COVID-19 prevention and control, health education, and prevention and health care. Of them, 27.01% were currently undertaking teaching work, and only 3.32% of them were conducting scientific research work related to general practice. The overall satisfaction of the trainees in the three theoretical training bases was above 90%, with no statistically significant difference among them (*P* > 0.05). Importantly, 84.11% of the followed-up personnel hoped to continue to participate in similar training in the future to improve their general practitioner core competences.

**Conclusion:**

The position transition training in Zhejiang Province has achieved good results, but the details of training and the implementation of policies in individual regions need to be improved. Most of the graduates were willing to continue their education, especially in general practitioners with special interests.

## Introduction

The demand for continuing education and professional development of medical doctors has often been strongly influenced by the characteristics of national and regional backgrounds. In recent years, China has been committed to strengthening the development of primary-level medical and health teams focusing on general practitioners, who play an important role in improving the health level of urban and rural residents and in reducing medical costs. To implement the spirit of The Guiding Opinions of The State Council on the Establishment of the General Practitioner System [1] and the Implementing Opinions of The General Office of The People’s Government of Zhejiang Province on Promoting the Construction of the Hierarchical Medical System [2], meeting the needs of grassroots health, fully enhancing teams of general practitioners, and promoting the implementation of the hierarchical medical system, the Health Commission of Zhejiang Province has carried out position transition training for general practitioners in the province since September 2017.

This position transition training has been intended mainly for on-the-job practicing doctors or assistant practicing doctors at the primary health institutions. They have been required to attend 1–2 years of job-transfer training to enhance the basic medical and public health service ability. The training has been conducted in standardized training bases for general practitioners recognized by the state (namely, tertiary general hospitals and qualified secondary hospitals as clinical training bases, and qualified community health service centers, township health centers and professional public health institutions as practice bases). At the end of the training, they can be registered as general practitioners or assistant general practitioners after passing the unified examination organized by the provincial health administrative departments and obtaining the certificate of qualification of general practitioners’ position transition training [1]. Position transition training is a transitional training for general practitioners that was launched nationwide in 2010 and implemented in accordance with the Training Outline for General Practitioners’ Position Transition Training in Primary Medical and Health Institutions (Trial) issued by the former Ministry of Health [3].

The position transition training in Zhejiang Province has been launched once a year, based on the method of full off-duty. Each training period lasts for 12 months, divided into three stages. The training contents include 15 days of theoretical training, 7 months of clinical comprehensive ability training, and 4 months of general practice ability training; different emphasis has been placed on the training modules [2]. The theoretical training base has been hosted by medical colleges A, B, and C, respectively, and the clinical practice has been hosted by medical institutions that have met relevant preset conditions. As of 2022, Zhejiang Province has completed four sessions of training. To understand the current situation of the trainees who have completed the training, we conducted a follow-up survey in March 2022.

## Objects and methods

### Respondents

By 2022, the position transition training in Zhejiang Province has completed four sessions,with a total number of 738. 253 personnel were selected by convenience sampling.

### Methods

Through online design by filling out a questionnaire investigation, the content includes the following aspects: whereabouts after the training, whether he/she has been registered as a general practitioner, whether to undertake general practice teaching and scientific research work, the current occupational environment, the improvement of post competence after receiving position transition training, willingness survey (such as the training program that they hope to participate in in the future), etc. Our teaching administrative personnel were responsible for the distribution, recycling, and checking of the questionnaire validity.

### Statistical analysis

All data were inputted twice by Epidata3.0 software to establish a database, and analyzed by Stata software. Enumeration data is expressed in frequency and percentage. The chi-square test was used to compare the groups, *P* < *0.05* (2-tailed test) was considered as statistically significant.

### Quality control

Through literature review and expert consultation, the questionnaire was designed to ensure the scientific nature of the questionnaire. After the questionnaire was confirmed, a preliminary test was carried out among some of the participants in the position transition training. The participants were informed about the purpose and importance of the survey so that active cooperation from respondents could be obtained. In the data processing stage, quality inspection was carried out to ensure that the questionnaire met the preset quality standards.

## Results

### Basic investigation information

All 253 participants completed the questionnaire with a recovery rate of 100%.

### Basic information of the followed-up personnel participating in training

Out of the followed-up 253 trainees, 93.68% (237) successfully completed the training and obtained the Training Certificate of General Practitioners. The number of participants in 2017, 2018, 2019, and 2020 accounted for 20.16%, 14.62%, 28.85%, and 36.36%, respectively. The theoretical training bases of A, B, and C medical colleges included 34.78%, 37.15%, and 28.06% of the training participants, respectively.

### General characteristics of the followed-up personnel

**Fig. 1:**
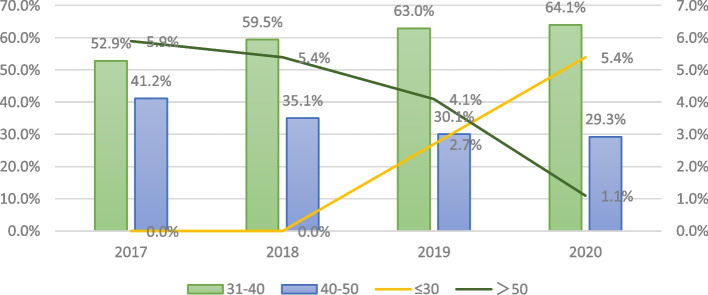
Age distribution of trainees in 2017–2020

**Fig. 2:**
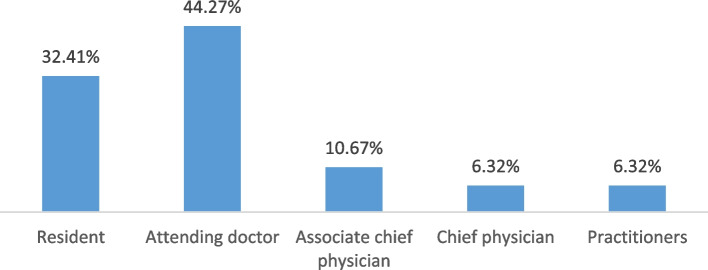
Current Professional title of the trainees

The male to female ratio was 1:1. Most of the participants were within 30–40 and 40–50 years old (61% and 33%, respectively). Residents and attending physicians accounted for 32.41% and 44.27%, respectively (Figs. 1 and 2). Currently, most of them were working in primary health care institutions(70.36%). Most had bachelor’s degrees (84%).

### The current occupational environment

**Fig. 3:**
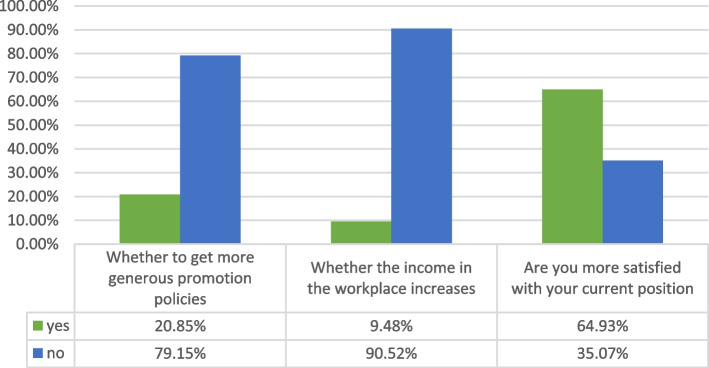
Changes after registration as a general practitioner

**Fig. 4:**
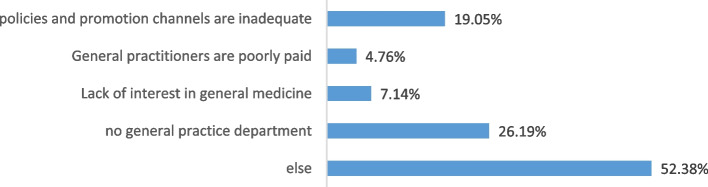
Reasons for not being registered as a general practitioner

The registered general practitioners constituted 83.4%, of which 82.94% added on the basis of the original registered scope of practice, whereas 17.06% changed their registration from the original registered scope of practice. Most physicians did not receive more favorable promotion policies or increased income after registered as a general practitioner, but more than half said they were more satisfied with their current positions than before (Fig. 3). Among the trainees, 16.6% were not registered as general practitioners, as can be seen in Fig. 4, and 52.38% (22) chose “other reasons”, as shown in Table 1. In reference to “If there is an opportunity to register in general practice, would you like to?”, 90.48% responded positively (“Yes”).

**Table 1:** Other reasons for not being registered as a general practitioner

Reason	Number
I haven’t had a health examination yet	2
Former major is major of union of traditional Chinese and western medicine, do not allow for registration for general medicine	1
Ready to register	2
Don’t let me register on the basis of the original registered scope of practice	1
Failed to pass the exam	6
Forget about it	1
My unit or department does not arrange registration	2
Registration of private clinics is conditional	1
The hospital is busy with COVID-19 and has not registered for me	1
Other	5

Among the 253 people surveyed, most of them have been engaged in medical work for more than five years, and only 25% have been engaged in general practice for more than five years.47.83% of them were engaged in internal medicine before attending the position transition training, and only 22.92% were engaged in general practice. After the training, approximately 70% of them were engaged in general practice.

**Fig. 5:**
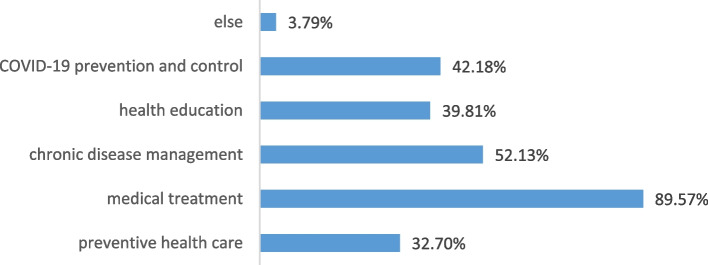
Main duties of current position

When asked their hospital’s recognition of general practice, the majority expressed “high recognition” or “recognition” of general practice (46.92% and 33.65%, respectively). After the training, approximately 80% of the trainees said residents were more satisfied with their services than before. Currently, the main duties they undertook were medical treatment, chronic disease management, COVID-19 prevention and control, health education, preventive health care and others (see Fig. 5), and the “others” include administrative work, temporary medical support and random tasks.

Out of the followed-up personnel, 27.01% were currently engaged in teaching work in their original work units, mainly teaching assistant general practitioners or “5 + 3” resident standardized training physicians, and 36.84% of them were also engaged in teaching undergraduate trainee or intern. The main teaching contents were clinical teaching, theoretical teaching, teaching management, and teaching evaluation.

Other 17.06% of the followed-up practitioners worked as hospital management personnel, such as the director, deputy director, department director, teaching director, deputy chief of medical department, community health service station master, and team leader. Only 3.32% of them were engaged in scientific research related to general practice. When asked if they are competent for their current job, 85.78% said they were competent, and 14.22% said they were partially competent.

### Self-evaluation of position transition training effect

**Fig. 6:**
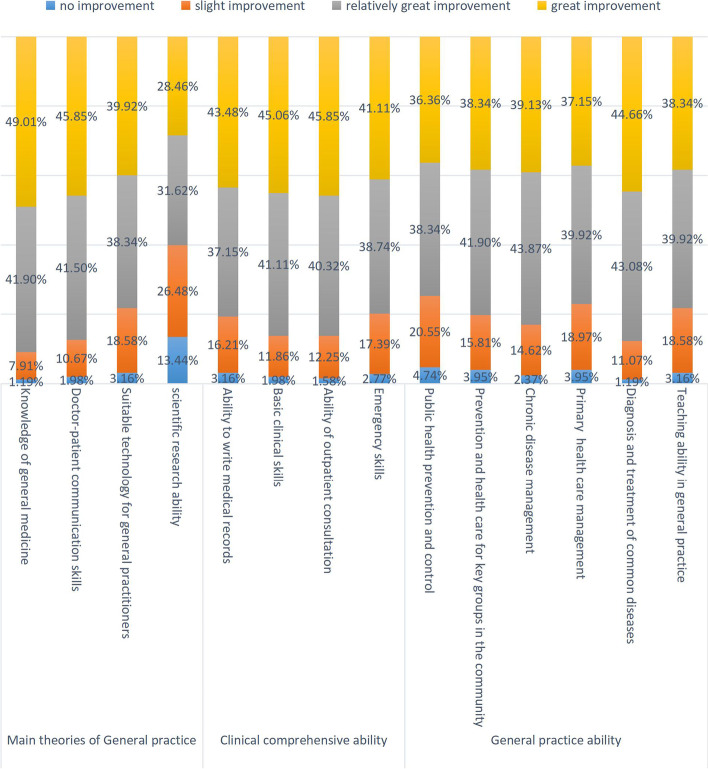
Self-evaluation of position transition training effect

The training content of position transition training has been divided into three parts: theoretical training, clinical comprehensive ability training, and general practice ability training, of which the theoretical training is face-to-face centralized training. In the clinical comprehensive ability training module, general practice department, internal medicine, pediatrics, emergency department, and gynecology are required rotation training departments, and the rest are elective rotation training departments. The general practice ability training is divided mainly into general practice and public health practice. We conducted a survey on the self-evaluation of the training effects of these three modules. The results showed that, on the whole, the training significantly improved after the training compared with before the training (Fig. 6). However, as can be seen from Fig. 6, approximately 40% of the trainees reported that their overall scientific research ability had not improved or had slightly improved.

The overall satisfaction of the trainees in the three theoretical training bases was above 90%, with no significant difference (*P* > 0.05). Some of the followed-up staff put forward valuable suggestions for the position transition training, such as low salary during training, difficult balance between work and study, imperfect relevant policies, poorly targeted training or irresponsible teaching, and registration problems (Table 2).

**Table 2:** Suggestions on the position transition training

Suggestions
One year is too short. Three years is recommended
Increase the learning time in outpatient department, increase practical operation and drill, and carry out online clinical first aid training
It is hoped that appropriate skills such as traditional Chinese medicine and first aid will be strengthened
I hope to have targeted training according to my own needs
I hope the teacher has a strong sense of responsibility
It is hoped that relevant authorities will implement preferential policies for general practitioners, improve the benefits of them, avoid drain of general practitioners
Accommodation is recommended
Doctors from different levels of hospital should be trained in different ways, so that doctors with different foundations can be promoted accordingly
Hope private clinic doctors can also be annotated as general practitioners
The shortage of staff in primary health care facilities has resulted in fewer doctors being able to go out for the training and it’s useless for us to get promoted
It is suggested to strengthen the training of general practitioners with special interests. General practitioners must master a core technology, or they will have no core competitiveness in hospitals

Approximately 75% of them said their autonomous learning ability had been greatly improved after the training. When they encounter clinical problems, most of them tend to consult literature(83.79%), refer to textbooks(79.45%) and consult colleagues(74.31%), and some of them rely on previous experience(49.01%) or search for answers on Baidu(45.85%).

### Survey of continuing education

Of all trainees, 15.02% also participated in other general practitioners training after the position transition training, such as senior teacher training, Zhejiang Cardiology sub-specialty training, traditional Chinese medicine general training, training of key general practitioners, general practitioners clinical diagnosis and treatment ability offline training, primary hypertension chief doctor training, disaster first aid training, British general practitioners training, etc. Nearly 85% responded that they would still be willing to participate in similar general training courses in the future. We also conducted a preliminary survey on the training programs that they hope to participate in, in which 84.11% of the followed-up personnel said that they hope to participate in the training of general practitioners with special interests in the future, and almost half of them hoped to have the opportunity to participate in the training of general practitioner teacher training and scientific research ability enhancement training.

## Discussion

As of 2022, Zhejiang Province has completed four sessions of general practitioners position transition training. Of the 253 followed-up trainees, 93.68% successfully completed their training and obtained the Certificate of General Practitioners Training. The majority of these doctors are young and middle-aged, and most of them have been engaged in medical work for a long time. Currently, most of them are working in grassroots health and medical institutions. Most of them had bachelor degree, and most of them had professional title of resident physician and attending physician, followed by associate chief physician and chief physician.

More than 80% of the doctors had registered their practice direction of general practice, most of which added on the basis of the original registered scope of practice, and a few changed their registration from the original registered scope of practice. Such a high registration rate is different from the survey results of Wu Tao [4] in 2012, which is due to the implementation of the general practitioner registration policy [5] and reflects part of the results of the position transition training in Zhejiang Province. At the time of this paper preparation, there were still a small number of doctors who had completed training but had not registered for general practice. The main reason was that the employer did not have general practice department and they were assigned to other specialties, general practice policies and promotion channels in individual regions or units that were not perfect, or they failed to pass the graduation examination. The survey found that more than 90% of them would be willing to register in general practice if these objective restrictions were removed.

Most units recognize the major of general medicine, and patients’ recognition of the doctors increases after the position transition training, which is similar to the research results of Wang Xiaolan [6]. Most of the physicians did not receive favorable promotion nor did their income increased after this registration, but the survey found that more than half of the physicians expressed more satisfaction with their current positions than with the earlier ones, indicating that preferential promotion policy of professional title or remuneration are not the only factors affecting job satisfaction, we suspect it has something to do with professional pride.However, the specific reasons need further investigation.The main duties of general practitioners after their registration as such include medical treatment, chronic disease management, COVID-19 prevention and control, health education, and preventive health care. It can be seen that general practitioners have heavy workload, which also reminds us to learn from the experience of other countries [7–13]. While vigorously cultivating general practitioners, we should establish an effective incentive mechanism and formulate preferential policies that can be implemented to improve the attractiveness of the posts and avoid the outflow of trained talents.

It is worth noting that nearly 50% of the followed-up personnel involved in the COVID-19 prevention and control, which indicates that post-transfer doctors play an important role in public health emergencies, which suggests that we can plan the prevention and control of infectious diseases into the key training programs of the position transition training in the future, so as to ensure that the primary health care system can effectively deal with the future epidemic of infectious diseases [14]. The novel coronavirus pneumonia epidemic has also exposed several problems in China’s grassroots medical talent team: 1) Grassroots general practitioners lack knowledge and practical experience of infectious disease prevention and control [15, 16]; 2) The lack of grassroots medical talents and high-level compound medical talents, and the structure of medical talents is unreasonable [17–19].The grass-roots health service system combined with prevention and treatment is the first line of defense for epidemic prevention or dealing with major public health events. The training program for general practitioners’ position transition obviously does not pay enough attention to or train them in this aspect. For example, in the clinical practice module, there is only one month’s learning requirement of respiratory department, and it is an elective major. Although reporting and handling of infectious diseases and public health emergencies are mentioned in the training module of grassroots general practice ability, it only takes one month to learn.In view of this situation, we suggest to increase the training of general practitioners on infectious disease knowledge and handling. Considering that some hospitals do not have infectious disease departments, they can jointly train doctors with local CDC.In addition, the position transition training of general practitioners is a good way to train high-level compound medical talents,most of the doctors participating in the training are specialists, who have been working in clinical practice for many years and have rich experience in diagnosis and treatment. Based on the requirements of the position transition training of general practitioners, we can conduct targeted training according to their interests and specialties or residents’ needs, so that they can serve residents to the greatest extent and save educational resources.

Generally speaking, various abilities have improved significantly after the training, but about 40% of the trainees still said that the scientific research ability has not improved or slightly, such problems also exist in the standardized training of general practice residents [20]. The possible reasons are as follows:Scientific research related to general practice is only involved in theoretical training, and after entering the clinical practice base, clinical work is the main focus, with very little time for scientific research training. In addition, The late start of the development of domestic General Practice, the lack of scientific sensitivity of the trainees, and the lack of strong scientific research ability of general practice teachers are also one of the reasons [21]. Scientific research plays an important role in the development of the discipline and should be paid more attention to.

Although the overall satisfaction of the training was very high, problems still existed, such as low salary during the training, difficult balance between work and study, imperfect relevant policies, poorly targeted training, and irresponsible teaching. These challenges also were reported to exist in other provinces in China [22].The application of the adult learning theory, with learner-centered emphasis has been considered a necessary condition for promoting deeper learning in medical education for decades [23, 24], For the problems mentioned by some followed-up personnel, such as “the training is not targeted, the training time is short, and the rotation is just a formality”, it is suggested that the training should pay more attention to the needs of trainees in the future. The requirements of doctors in different regions or medical institutions may be different. For example, a study in Ireland [25] found that the number of general practitioners who chose pre-hospital emergency care as the theme of continuing education was almost twice that of urban general practitioners.

For doctors, autonomous learning ability is very important, which can reflect a person’s lifelong learning or problem-solving ability. Especially for general practitioners, learning the latest medical knowledge is more important [26], and we also conducted a survey on this. It was gratifying to see that more than half of them (75%) said that their autonomous learning ability was significantly improved after training.

Most of the follow-up personnel hoped to have the opportunity to participate in the training of the general practitioners with special interests in the future. A general practitioner with special interests training is thought to have originated in the United Kingdom and is intended to reduce the burden of specialists in certain fields and provide better services to patients [27–29]. The services provided to patients by such general practitioners are considered superior to those provided by non-general practitioners, and in the current environment, the cultivation of general practitioners with special interests can make more effective use of scarce health resources [30].

## Thoughts and suggestions

Since the position transition training of general practice was launched in Zhejiang Province, our team has been fully involved in this training program and has accumulated certain experience. Combined with the results of this survey, we also have some thoughts and suggestions.

All institutions should implement policies related to the training and use of incentive mechanisms for general practitioners, and include the implementation of such policies into the performance assessment of governments at all levels [31]. For areas that fail to be implemented, field visits should be made to understand the reasons and targeted solutions should be provided.

Scientific research is the weak link of general practitioners, and the improvement of scientific research ability is one of the key factors to promote general practitioners to carry out scientific and standardized disease prevention and treatment. Currently, there are scientific research courses on theoretical content, but with little practical effect and insignificant directions on how to really improve the potential for scientific research. Hence, these weak points need to be addressed in the future.Zhong Li [32] put forward several suggestions for this, which can be used as reference: to increase the trainees’ and teachers’ attention to scientific research and innovation,in addition to improving trainees’ theoretical knowledge and clinical skills, we should also pay attention to improving their scientific research ability.In the current three-stage training mode of “teaching in medical colleges + rotation in teaching hospitals + practice in grassroots units”, various resource advantages in each stage should be given full play to create good conditions for scientific research training for trainees. It is difficult to improve scientific research ability in a short period of time, so we can refer to foreign experience in continuing medical education [33]. For example, after the general practitioners who have been transferred back to their original units, they still have the opportunity to voluntarily participate in the study of scientific research ability, which can be carried out in the form of meeting or inviting experts.

Some of the doctors(27.01%) returned to their original units to undertake teaching work to alleviate to a certain extent the shortage of teachers in general practice at the grass-roots level. Future training should pay more attention to teaching ability, especially in the teaching hospitals. When accepting doctors participating in the position transition training, we should know in advance whether they would undertake teaching work in the future. Then, we should pay attention to the improvement of the teaching ability of doctors who would become teachers in the future.

In recent years, public health events have been occasionally held in China, and a new role has been performed by primary-level medical institutions. The survey found that nearly 50 percent of trained doctors have been involved in COVID-19 prevention and control. The skill training of general practitioners in dealing with public health emergencies should be an important part of the training.

84.11% of the follow-up staff expressed the hope that they could participate in sub-specialty ability training of general practice in the future, so the continuing education of general practitioners should not be ignored. Online and offline continuing education programs can be held regularly to ensure the sustainable development of the profession of general practitioners and constantly improve the core competitiveness of general practice. In terms of the content of continuing education, disciplinary barriers should be broken, especially the cultivation of basic public health service ability of grass-roots general practitioners, and the cultivation of clinical medicine and public health talents and the construction of talent team should be strengthened [34].

## Conclusion

As one of the important ways to train general practitioners in China, the exploration of the position transition training of general practitioners has been ongoing, such as the trainer needs, evaluation of training effects, etc. The training of general practitioners in Zhejiang Province has achieved good results, but the details of training and the implementation of policies in individual regions need to be improved. Hopefully, this investigation can provide some reference for the training of general practitioners.

## Strengths and limitations

In China, there are few investigations on the status quo of work after completion of the position transition trainingof general practitioners, especially in Zhejiang Province. Since Zhejiang launched the training program in September 2017, this is the first study of this kind.Since there are few similar training methods for general practitioners abroad, this study can provide a certain reference for foreign counterparts to understand the training mode of Chinese general practitioners.This survey found some problems and even interesting phenomena in the position transition training of general practice, which is worth further research in the future.

This study had the following limitations. Convenience sampling was adopted in this survey, and non-random sampling method may lead to some selection bias. Therefore, the results of this survey may not accurately reflect the situation of the whole province. Moreover, we used 4-scale self-evaluation questionnaire to evaluate the improvement of GPs’ ability. The reliability needs to be tested further.

## Data Availability

The datasets generated and/or analysed during the current study are not publicly available due to privacy or ethical restrictions. but are available from the corresponding author on reasonable request.
